# Spatial distribution of the time between diagnosis and treatment of breast cancer among women in Brazil

**DOI:** 10.1371/journal.pone.0361469

**Published:** 2026-10-28

**Authors:** Paulo Vitor de Souza Silva, Letícia Gabriella Souza da Silva, Ana Camily da Costa Cruz, Amarildo Gomes Cavalcante Júnior, Ephraim Phlorencio da Costa Júnior, José Levi Araújo Dutra, Nayara Priscila Dantas de Oliveira, Isabelle Ribeiro Barbosa, Dyego Leandro Bezerra de Souza

**Affiliations:** 1 Graduate Program in Collective Health, Federal University of Rio Grande do Norte - UFRN, Natal, Rio Grande do Norte, Brazil; 2 Federal University of Rio Grande do Norte - UFRN, Natal, Rio Grande do Norte, Brazil; 3 Graduate Program in Rehabilitation and Functional Performance, Physiotherapy Department, University of Pernambuco - UPE, Petrolina, Pernambuco, Brazil; 4 Graduate Program in Collective Health, Trairi Faculty of Health Sciences, Federal University of Rio Grande do Norte - UFRN, Natal, Rio Grande do Norte, Brazil; 5 Graduate Program in Collective Health, Department of Collective Health, Federal University of Rio Grande do Norte - UFRN, Natal, Brazil; Tangshan People's Hospital, CHINA

## Abstract

Female breast cancer presents high incidence and mortality rates and is considered a significant public health problem. The timeliness of oncological treatment is a fundamental factor in the prognosis and survival of patients. The objective of this study was to analyze the spatial distribution pattern of the percentage of women who initiated breast cancer treatment within 60 days and its association with socioeconomic indicators and the availability of health services in Brazil. An ecological study was conducted using 133 Intermediate Regions of Urban Articulation (IRUA), based on records from the period 2013–2019. Data on the percentage of treatment initiation within 60 days were obtained from the Hospital Cancer Registry Integrator (IHCR). Socioeconomic variables were collected from the Atlas of Human Development in Brazil and the Institute for Applied Economic Research (IPEA). Data on the availability and density of health professionals and services were obtained from the National Registry of Health Establishments (NRHE) and the Hospital Information System (HIS). The Global Moran’s Index and the Local Indicator of Spatial Association (LISA) were used to verify the existence of territorial clusters. Multivariate analysis employed models with global spatial effects. The percentage of breast cancer cases that initiated treatment within 60 days was 48.9% (95% CI: 48.6% − 49.1%), with the highest adherence observed in the IRUA of Cascavel (85%) and the lowest in the IRUA of Rio de Janeiro (16.6%). In the final Spatial Lag Model, positive associations were observed with the density of Family and Community Physicians (β = 0.35; p = 0.007) and the proportion of surgical procedures (β = 0.30; p = 0.001). The analysis indicates that compliance with the 60-day law is related to the structure and organization of health services, highlighting the importance of primary health care as the coordinator of care and the role of surgical capacity in the spatial distribution pattern.

## Introduction

Female breast cancer is the most frequently diagnosed malignancy and the leading cause of cancer-related death among women worldwide [1,2]. In Brazil, it also has the highest incidence among all cancer types, with important regional differences associated with inequalities in exposure to risk factors, prevention, early detection, and timely access to treatment [3,4].

Timely initiation of cancer treatment is a key determinant of prognosis among women with breast cancer [5,6]. In Brazil, Law 12,732, enacted in 2012, established that patients with a confirmed diagnosis of malignant neoplasia have the right to begin treatment within 60 days after histopathological confirmation of the diagnosis, representing a milestone in guaranteeing access to cancer care [7].

Despite this legal framework, delays in the initiation of breast cancer treatment remain frequent. Previous studies have reported waiting times ranging from 63 to 206 days between diagnosis and treatment initiation [8–11]. Delays in treatment initiation may directly compromise clinical outcomes, as women who receive timely treatment tend to achieve better survival outcomes [5,6].

Access to cancer treatment is influenced by socioeconomic factors and the organization of health services, particularly the availability of healthcare professionals and specialized services. In a country marked by substantial regional inequalities, differences in the distribution of these resources may compromise access to treatment and compliance with the legally established timeframe [10,11].

Although previous studies have investigated delays in breast cancer treatment and their associated factors, few have assessed compliance with the 60-Day Law while simultaneously considering the spatial distribution of cases, socioeconomic inequalities, and the availability of health services across Brazilian territories. Understanding these territorial patterns may contribute to identifying priority areas for intervention and improving the organization of oncology care networks.

Therefore, the aim of this study was to analyze the spatial distribution pattern of the percentage of women who initiated breast cancer treatment within 60 days and to determine its association with contextual socioeconomic indicators and the availability of health services in Brazil.

## Methods

### Study design and population

This is an observational ecological spatial analysis study that used the 133 Intermediate Regions of Urban Articulation (IRUA) defined by the Brazilian Institute of Geography and Statistics (IBGE) for the year 2017 [12].

The IRUA constitute an intermediate territorial scale between the Brazilian states (federative units) and the Immediate Geographic Regions of Urban Articulation. Each of these regions comprises a group of municipalities that share common characteristics and relationships in the provision of goods and services, including health services, thereby providing a more detailed representation of the Brazilian territory [12].

The decision to use the IRUA as the territorial unit of analysis, based on the 2017 reference framework, was grounded in its ability to represent urban articulations and the contextual realities of the period evaluated in this study.

Analytical cases of malignant breast neoplasm (C50) were included in the study, classified according to the 10th revision of the International Classification of Diseases, 10th Revision (ICD-10) [13]. Eligible cases included women aged 20–99 years, diagnosed between 2013 and 2019, and treated in hospital-based oncology care units in Brazil. Only cases between 20–99 years of age were considered, according to the current classification that defines childhood and adolescent cancers as those occurring up to 19 years of age [14].

The following cases were excluded from the study: carcinoma in situ (TNM 0), records without information on the state and municipality of residence of the patient, cases with a previous history of oncological treatment, and cases lacking data regarding diagnosis and treatment (dates of first diagnosis and treatment initiation, as well as the type of first oncological treatment).

### Variables and data sources

The outcome analyzed was the time in days elapsed between the first diagnosis and the initiation of oncological treatment. This time interval was dichotomized into “up to 60 days” and “more than 60 days,” considering compliance with Federal Law 12,732/12 [7].

Data from 2013 to 2019 were aggregated by IRUA. The outcome was defined as the percentage of women with breast cancer who initiated treatment within 60 days among all eligible cases recorded during the study period in each IRUA.

Data on the time interval between diagnosis and treatment were obtained from the Hospital Cancer Registry Integrator (IHCR). The HCR integrator consolidates hospital data from Hospital Cancer Registries across the 26 Brazilian states and the Federal District [15]. The data were accessed for research purposes on march 25, 2024. The dataset was publicly available and contained no information that could identify individual participants.

Within the HCR system, cancer cases are recorded as analytical cases, which involve diagnosis and treatment performed at the reporting institution, and non-analytical cases, which include cases that arrive at the hospital already treated, cases diagnosed through necropsy, or cases with no therapeutic alternatives [16].

The case registration procedure may also be classified according to diagnostic and treatment pathways. Cases without prior diagnosis refer to patients who arrive at the hospital unit without previous diagnostic investigation and undergo both diagnosis and treatment at the same institution. Cases with prior diagnosis include patients referred to the oncology unit with an established diagnosis, for whom only treatment is initiated at that institution [16].

The independent variables in this study were population indicators related to socioeconomic characteristics and the availability of health services within each IRUA. The selection of independent variables was theoretically supported by the Andersen Behavioral Model of Health Services Use. According to this model, the use of health services is influenced by three main dimensions: predisposing characteristics, enabling factors, and health needs [17,18].

The contextual variables adopted in this analysis relate to resources available within the territory that may facilitate or restrict access, such as health infrastructure and the availability of professionals and specialized services. In addition, socioeconomic indicators reflect the social structure, considered an element that conditions both the resources available to the population and their opportunities for access to health services [17–19].

Socioeconomic indicators, including the Social Vulnerability Index (SVI), the Gini Index, population aging rates, and female illiteracy rates, were obtained from the Atlas of Human Development in Brazil, made available by the United Nations Development Programme (UNDP) [20], and from the Institute for Applied Economic Research (IPEA) [21], based on data from the year 2010. These indicators were collected at the municipal level and subsequently aggregated by IRUA, weighted by population.

Data on the availability and density of health professionals and services were obtained from the National Registry of Health Establishments [22] and the Hospital Information System of the Brazilian Unified Health System [23]. Specific indicators were calculated for the year 2016. For the calculation of these indicators, population data from the 2010 Demographic Census, as well as municipal population counts and estimates by municipality, sex, and age, produced by the IBGE, were used as denominators [24]. Table 1 presents the characterization of the dependent and independent variables included in the study.

**Table 1:** Detailed description of the dependent variable and the independent variables used in the spatial pattern analysis.

Variable			Source	Description
**Dependent**	**Time to Treatment Initiation**	**Time to Oncological Treatment Initiation**	HCR Integrator (2013-2019)	Time interval in days between the date of the first tumor diagnosis and the initiation of oncological treatment at the hospital care unit.
**Independent (Contextual)**	**Socioeconomic**	**Gini Index**	Atlas of Human Development in Brazil (2010)	Measures the degree of inequality in the distribution of individuals according to per capita household income.
		**Social Vulnerability Index (SVI)**	IPEA (2010)	Calculated as the arithmetic mean of the following sub-indices: Urban Infrastructure SVI, Human Capital SVI, and Income and Labor SVI.
		**Population Aging Rate**	Atlas of Human Development in Brazil (2010)	Ratio between the population aged 65 years or older and the total population, multiplied by 100.
		**Female Illiteracy Rate**	Atlas of Human Development in Brazil (2010)	Ratio between the female population aged 18 years or older who are unable to read or write a simple note and the total number of women in this age group, multiplied by 100.
**Independent (Contextual)**	**Availability and Density of Health Professionals and Services**	**Density of Oncology Specialist Physicians**	National Registry of Health Establishments – CNES (2016)	Ratio between the average number of oncology specialist physicians registered in the CNES and the total population for that year, multiplied by 1,000,000, by IRUA.
		**Density of Family and Community Physicians**		Ratio between the average number of family and community physicians registered in the CNES and the total population for that year, multiplied by 1,000,000, by IRUA.
		**Density of Health Facilities Specialized in Oncology**		Ratio between the average number of health facilities accredited for oncology care registered in the CNES and the total population for that year, multiplied by 1,000,000, by IRUA.
		**Proportion of Surgical Procedures for the Treatment of Breast Neoplasms**	Hospital Information System -SIH (2016)	Ratio between the average number of breast oncology surgical procedures and the total female population for that year, multiplied by 100, by IRUA.

### Data analysis

Initially, a descriptive analysis of the data was performed using GeoDa software, version 1.22, employing the IRUA as the spatial unit for the creation of thematic maps. This analysis enabled the spatial description of the distribution of the percentage of breast cancer cases that initiated treatment within 60 days.

To verify the existence of territorial clusters, Global Moran’s I was used, which allows the identification of areas with their own spatial dynamics. The Local Indicator of Spatial Association (LISA) [25] was adopted to identify significant patterns of spatial correlation based on first-order neighbors. For this analysis, first-order queen contiguity was used.

According to the significance level of LISA, the IRUA were classified as positive spatial correlation when regions had neighboring areas with similar values (high-high and low-low), and negative spatial correlation when neighboring regions presented distinct values (high-low and low-high).

Prior to multivariate spatial modeling, a bivariate analysis was conducted between the dependent variable and the contextual indicators. The normality of the variables was assessed using the Shapiro-Wilk test. Pearson correlation coefficient was applied to variables with a normal distribution, whereas Spearman rank correlation coefficient was used for variables with a non-normal distribution. Correlation coefficients and their corresponding p-values were considered to support the selection of candidate variables for spatial modeling, together with theoretical and epidemiological plausibility criteria.

Spatial dependence diagnostics were initially assessed using the residuals from the ordinary least squares (OLS) model, including Moran's I, Lagrange Multiplier (LM) tests for spatial lag and spatial error dependence, and their robust counterparts. Spatial Lag (SLM) and Spatial Error (SEM) models were subsequently estimated and compared using log-likelihood, Akaike Information Criterion (AIC), Bayesian Information Criterion (BIC), and R². The final model was selected based on the joint assessment of spatial dependence diagnostics, model fit, and the substantive specification of spatial dependence in the outcome.

For the multivariate analysis, a global spatial effect model was adopted using the Spatial Lag model. The statistically significant variables in the bivariate analyses, as well as those with theoretical plausibility for inclusion, were kept in the multivariate model.

The residuals generated by the Spatial Lag model were analyzed using Global Moran’s I and the data dispersion histogram to verify the elimination of spatial autocorrelation after the execution of the multivariate statistical model. Additionally, residual normality was assessed using the Jarque–Bera test and visual inspection of the quantile–quantile (Q-Q) plot. The statistical models and the calculation of Global Moran’s I and LISA were performed using GeoDa software version 1.22.

The study used secondary, non-identified, publicly available, and unrestricted-access data, and therefore ethical review by a Research Ethics Committee was not required, in accordance with Resolution 510/2016 of the National Health Council of the Brazilian Ministry of Health [26], in conjunction with Resolution 466/2012 [27].

## Results

The study included 192,392 breast cancer cases between 2013 and 2019. The annual proportion of treatment initiation within 60 days ranged from 52.2% in 2013 to 43.3% in 2019 (S2 Table). The number of cases contributing to the regional estimates varied across the 133 IRUAs (S3 Table). The distribution of cases according to year and IRUA is presented in S4 Table.

In Brazil, the proportion of cases that initiated oncological treatment in compliance with the 60-Day Law was 48.9% (95% CI: 48.6%−49.1%). The percentage of compliance with the legislation varied across regions, with the lowest adherence observed in the IRUA of Rio de Janeiro (16.6%) and the highest in Cascavel (85%).

Fig 1 presents the spatial distribution of the percentage of compliance with the law (treatment initiation within 60 days) across the 133 Intermediate Regions of Urban Articulation during the period 2013–2019.

**Fig 1:**
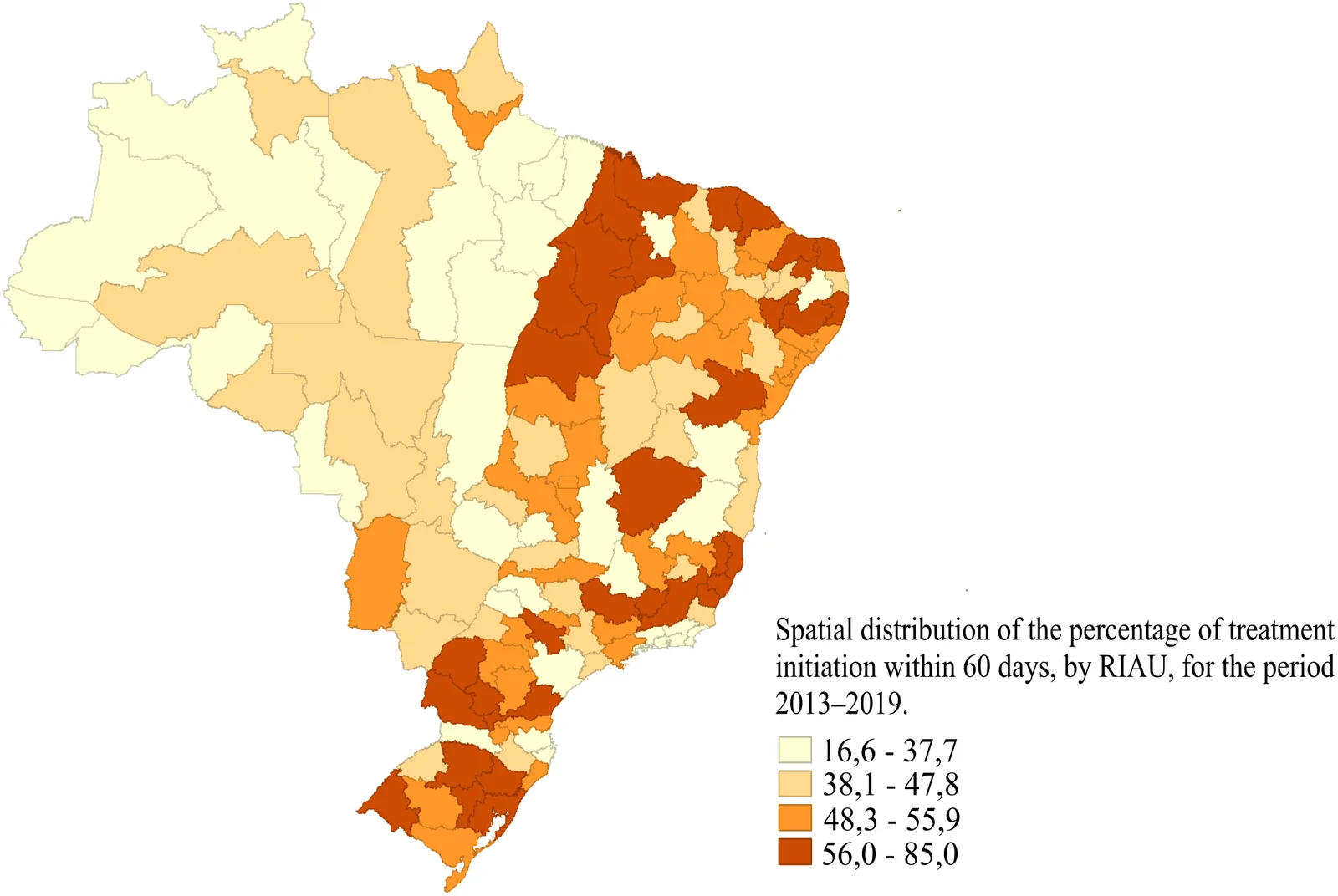
Spatial distribution of the percentage of treatment initiation within 60 days in the IRUA, 2013-2019.

The presence of spatial autocorrelation for the percentage of treatment initiation within 60 days was confirmed using Global Moran’s I (I = 0.291; p = 0.001). Based on the local LISA statistic, the IRUA were classified according to the level of statistical significance. Fig 2 shows the spatial autocorrelation analysis of the percentage of compliance with the legislation across the Brazilian territory.

**Fig 2:**
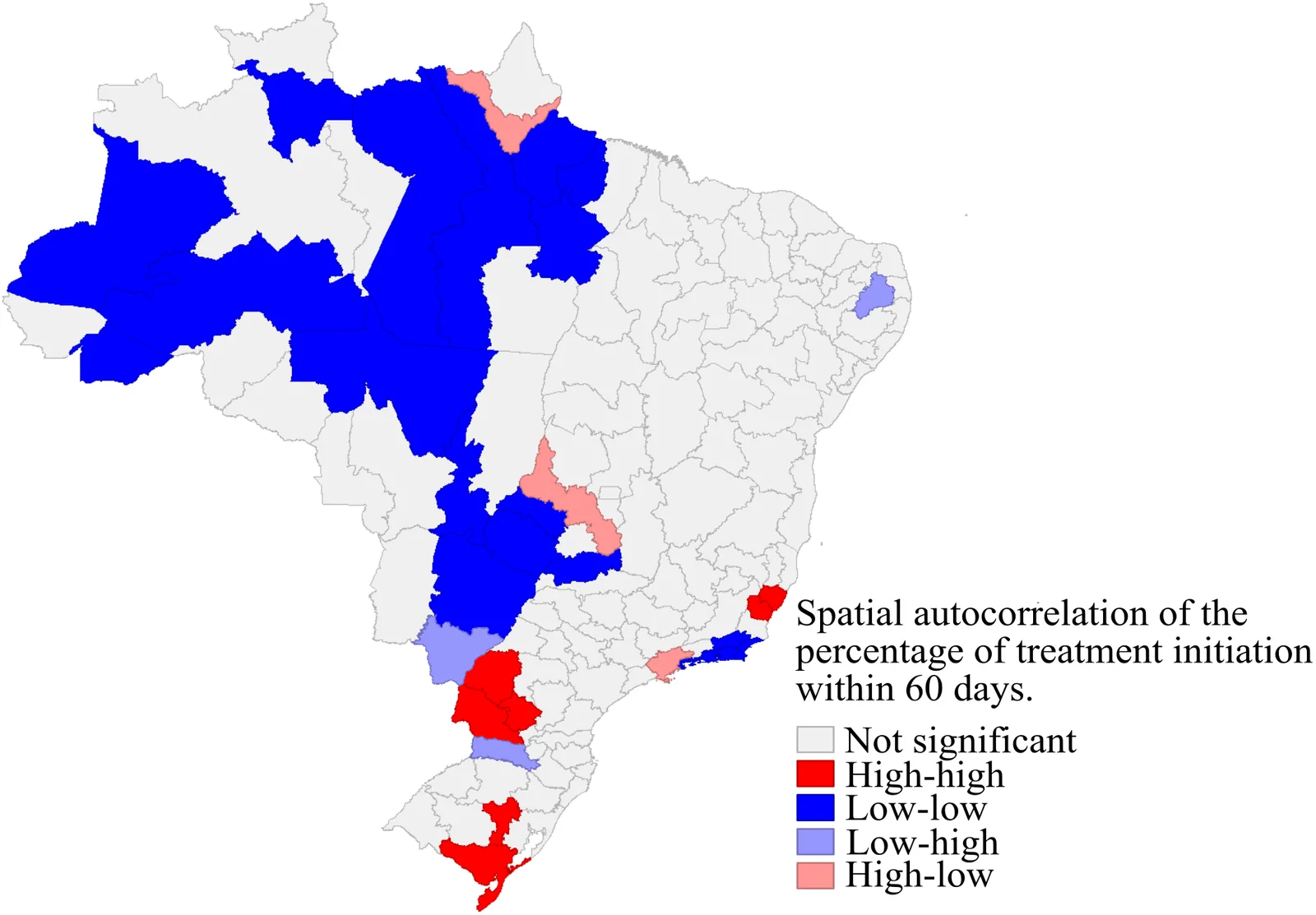
Spatial distribution of clusters of treatment initiation within 60 days in Brazil, by IRUA, 2013-2019. Moran’s I = 0.291; p = 0.001.

Fig 3 shows the bivariate spatial association between the percentage of treatment initiation within 60 days and the socioeconomic and health service availability indicators. Maps and Moran scatterplots present the results of the bivariate Moran’s I analysis. Pearson (*r*) or Spearman (*ρ*) correlation coefficients and corresponding p-values are also shown to facilitate comparison with conventional bivariate association measures.

**Fig 3:**
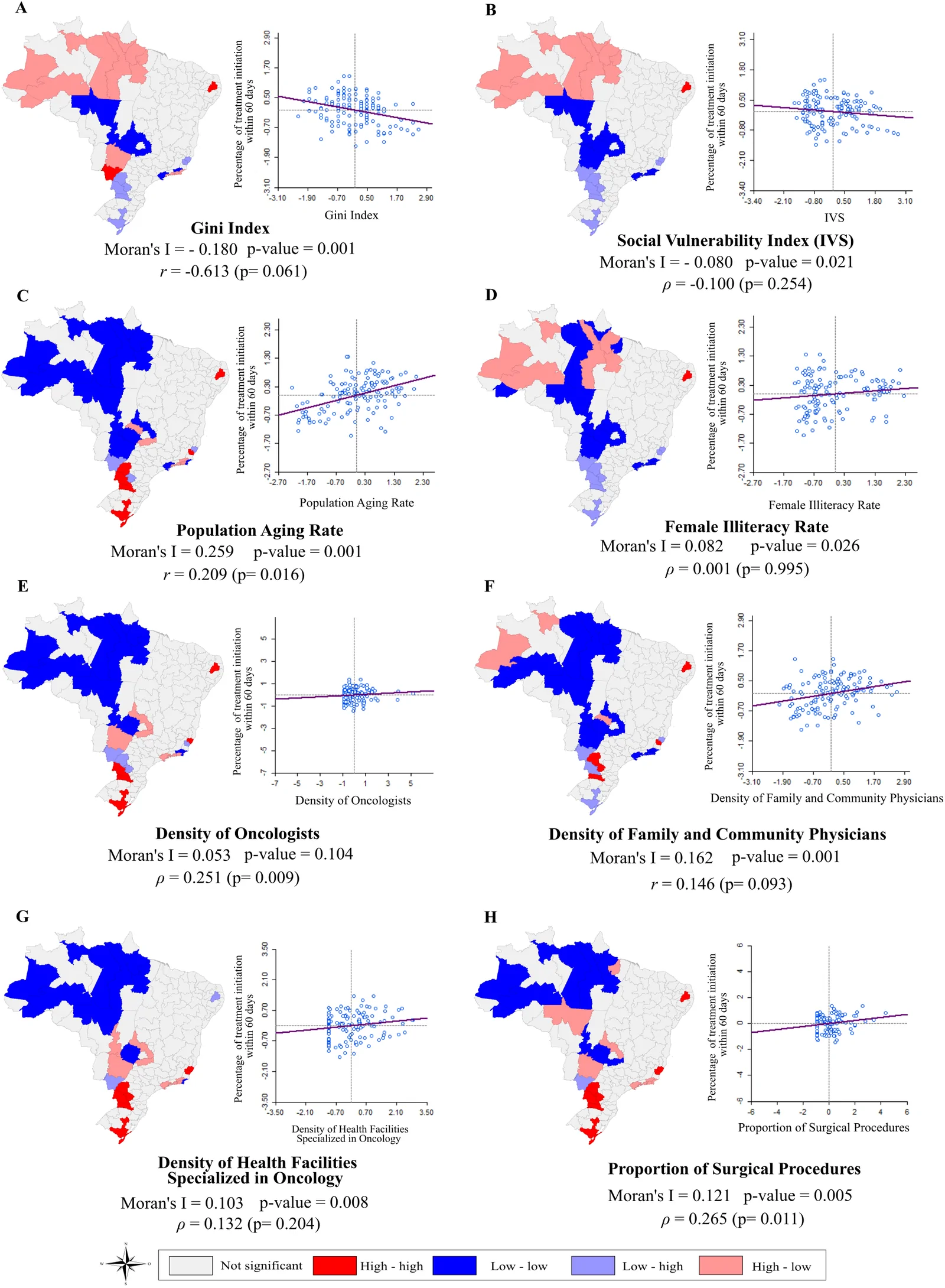
Bivariate spatial association between the percentage of treatment initiation within 60 days and socioeconomic and health service availability indicators in Brazil, by IRUA, 2013-2019.

A predominance of positive spatial associations was observed, except for the Gini Index (Moran’s I = −0.180; p = 0.001) and the Social Vulnerability Index (Moran’s I = −0.080; p = 0.021), which exhibited negative spatial autocorrelation. The variable “Density of Oncologists” did not show statistically significant spatial autocorrelation in the bivariate Moran analysis (Moran’s I = 0.053; p = 0.104). Pearson and Spearman correlation coefficients and their respective p-values are also presented in Figure 3 to allow comparison between conventional bivariate association measures and the spatial association patterns identified by Moran’s I.

The multivariate spatial regression model analyzing the percentage of treatment initiation within 60 days showed an explanatory power of 32.12%.

Based on the joint assessment of the spatial diagnostics, model fit, and substantive specification of spatial dependence in the outcome, the SLM was retained as the final model. In the final SLM, the spatial autoregressive coefficient was positive and statistically significant (ρ = 0.409; SE = 0.096; z = 4.258; p < 0.001). The likelihood ratio test for spatial lag dependence was also statistically significant (LR = 13.662; p < 0.001).

The model residuals showed a normal distribution, and Global Moran’s I was 0.001 (p = 0.431). The Jarque-Bera test yielded a value of 0.850 (p = 0.654), indicating no significant deviation from normality, and visual inspection of the quantile–quantile (Q-Q) plot corroborated this result, showing that the residuals closely followed the theoretical normality line. Table S1 presents the comparison between the tested models, and S1 Fig presents the analysis of the residuals.

Table 2 presents the results of the spatial regression analysis by IRUA. The final model included the variables density of family and community physicians, proportion of surgical procedures, and density of accredited oncology facilities. All regression coefficients were positive. However, statistical significance was observed only for the density of Family and Community Physicians and the proportion of surgical procedures.

**Table 2:** Multivariable spatial regression analysis of the percentage of breast cancer treatment initiation within 60 days and its association with health service availability indicators in Brazil, by IRUA, 2013-2019.

	Coefficient β	Standard Error	z	p-value	95%CI
***Population indicators of health service availability***					
Density of Family and Community Physicians	0.35	0.13	2.65	0.007*	0.092–0.615
Proportion of Surgical Procedures	0.30	0.08	3.63	0.001*	0.143–0.477
Density of Health Facilities Specialized in Oncology	0.48	0.66	0.720	0.465	−0.820–1.791

*statistically significant; z: test statistic; CI: confidence interval.

**Spatial parameter:** ρ = 0.409; SE = 0.096; z = 4.258; p < 0.001.

**Model fit:** R^2^ = 0.321; Log-likelihood = −514.43; Criterion de Akaike = 1038.86; Criterion de Schwarz = 1053.31

The inclusion of the density of accredited oncology facilities was justified by its theoretical plausibility, given its relevance to access to specialized oncology care. The density of family and community physicians was retained because its inclusion improved the overall performance and fit of the final model.

In the multivariable Spatial Lag Model, the density of family and community physicians (β = 0.35; 95% CI: 0.092–0.615; p = 0.007) and the proportion of surgical procedures (β = 0.30; 95% CI: 0.143–0.477; p = 0.001) were positively and statistically significantly associated with the percentage of treatment initiation within 60 days. The density of health facilities specialized in oncology showed a positive coefficient (β = 0.48; 95% CI: −0.820–1.791), but the association was not statistically significant (p = 0.465).

## Discussion

The findings of this study revealed territorial variation in compliance with the Brazilian legislation that establishes the initiation of cancer treatment within 60 days after diagnosis. Less than half of the analyzed breast cancer cases initiated treatment within the legally established timeframe, highlighting regional inequalities in timely access to healthcare services. Furthermore, the identification of spatial clusters indicates that compliance with the legislation is not homogeneously distributed across the Brazilian territory, suggesting the influence of contextual factors related to the organization of health services.

The spatial analysis identified a positive association between the density of Family and Community Physicians and the percentage of treatment initiation within 60 days. This finding represents an association at the territorial level and does not establish that greater physician availability directly causes shorter treatment delays. Nevertheless, the observed association is consistent with the potential role of Primary Health Care (PHC) as a strategic component in the organization of cancer care networks. PHC plays a fundamental role in identifying suspicious signs and symptoms, ensuring timely referral for diagnostic investigation, and coordinating care throughout the healthcare pathway [28–30].

Strengthening PHC has been recognized as an essential strategy for improving access to breast cancer diagnosis and treatment. The work of family health teams facilitates the identification of population health needs, the organization of screening activities, and the articulation with other levels of care, contributing to the reduction of access barriers and the optimization of care pathways [31–33].

In addition to facilitating access to specialized services, PHC plays an important role in care coordination and continuity of care for women with breast cancer. The integration between primary and specialized care promotes a clearer definition of responsibilities across different levels of care and contributes to greater efficiency within healthcare networks, reducing delays and improving health outcomes [34–37].

Another relevant finding was the positive association between the percentage of treatment initiation within 60 days and the proportion of surgical procedures performed for breast cancer treatment. This territorial-level association may be related to differences in the organization and availability of specialized healthcare services. However, given the ecological design of the study, this finding should not be interpreted as evidence that greater surgical availability directly causes more timely treatment initiation.

Evidence from Brazilian studies has shown that patients undergoing surgery are more likely to initiate treatment within the legally established timeframe when compared with those referred to therapeutic modalities that depend on more complex services, such as chemotherapy and radiotherapy [9,38]. This finding may be partially explained by the diagnostic process itself, since histopathological confirmation is sometimes established after the surgical intervention, thereby reducing the officially recorded interval between diagnosis and treatment initiation [38,39].

Conversely, the need to travel to specialized chemotherapy or radiotherapy centers may represent an additional barrier to timely treatment. The concentration of these services in large urban centers and the unequal distribution of oncology infrastructure across the Brazilian territory contribute to longer waiting times and lower compliance with the current legislation [9,10,40].

Although socioeconomic indicators were associated with the outcome in the bivariate analysis, these variables were not retained in the final model. Nevertheless, the findings should be interpreted within the Brazilian context, which is characterized by profound social and territorial inequalities. Previous evidence indicates that socioeconomic factors influence access to healthcare services, particularly specialized care, creating unequal opportunities for diagnosis and treatment across different regions of the country [41–43].

These inequalities become particularly evident when examining migratory flows related to cancer treatment. Recent studies have demonstrated that patients residing in areas with limited availability of specialized services frequently need to travel to other municipalities or states to receive care, especially in the North and Northeast regions of Brazil [44]. This process imposes additional financial, social, and emotional burdens on patients and may contribute to delays in treatment initiation.

The findings of this study reinforce the importance of the structure and organization of oncology care networks for compliance with Brazilian legislation. Strategies aimed at strengthening PHC, expanding the capacity of specialized services, and reducing regional inequalities may contribute to improving timely access to treatment and reducing the disparities observed across territories [37,44–46].

Some limitations of this study should be acknowledged. As an ecological study, the findings reflect associations observed at the territorial level and do not allow individual-level inferences, being subject to ecological fallacy. The data sources refer to different time periods. These temporal differences should be considered when interpreting the observed associations.

The Hospital Cancer Registries include only cases from participating hospital-based registries and, therefore, may not represent all women diagnosed with breast cancer in Brazil. The findings should be interpreted considering the coverage and characteristics of this data source.

The number of eligible cases varied across IRUAs, resulting in different denominators for the regional percentages. Thus, estimates from regions with smaller numbers of cases may be less precise than those from regions with larger denominators.

Residual confounding is also possible, as regional characteristics not included in the analysis may be associated with timely treatment initiation. In addition, the final spatial model captured part of the regional variation in timely treatment initiation (R^2^ = 0.321), suggesting that other unmeasured contextual factors may influence the outcome.

Finally, the study period ended in 2019 and, therefore, did not capture potential changes in access to cancer diagnosis and treatment associated with the COVID-19 pandemic. Despite these limitations, the use of nationwide databases and spatial analytical techniques made it possible to identify relevant territorial patterns associated with compliance with the 60-Day Law in Brazil.

## Conclusions

The spatial analysis identified territorial variation in the percentage of breast cancer treatment initiation within 60 days in Brazil. Higher densities of Family and Community Physicians and higher proportions of surgical procedures were positively associated with the percentage of timely treatment initiation across the analyzed regions.

These findings suggest that regional differences in timely treatment initiation may be related to contextual characteristics of health service availability and organization, particularly those involving primary health care and surgical procedures. Compliance with the 60-Day Law may therefore be associated with organizational characteristics of the Brazilian health system.

## Supporting information

S1 Table Comparison of regression models for the percentage of breast cancer treatment initiation within 60 days. (DOCX)

S2 Table Distribution of included breast cancer cases and treatment initiation within 60 days by year, Brazil, 2013–2019. (DOCX)

S3 Table Distribution of included breast cancer cases and treatment initiation within 60 days by Intermediate Region of Urban Articulation, Brazil, 2013–2019. (DOCX)

S4 Table Distribution of included breast cancer cases by year and Intermediate Region of Urban Articulation, Brazil, 2013–2019. (DOCX)

S1 Fig Residual analysis of the Spatial Lag model. (TIF)
