# Supplementary material for: Spatial distribution of the time between diagnosis and treatment of breast cancer among women in Brazil
Source: PLoS One. 2026 Oct 28;21(10):e0361469. doi: 10.1371/journal.pone.0361469 (PMC13716208; doi:10.1371/journal.pone.0361469)
Supplement: Supplementary file 1 [file pone.0361469.s001.docx]

S1 Table. Comparison of regression models for the percentage of breast cancer treatment initiation within 60 days.

| **Model** | **Log Likelihood** | **AIC** | **BIC** | **R²** |
| --- | --- | --- | --- | --- |
| Spatial Lag Model | -514,43 | 1038,86 | 1053,31 | 0,3212 |
| Spatial Erro Model | -515,38 | 1038,78 | 1050,34 | 0,3152 |
| Ordinary Least Squares (OLS) | -521,26 | 1050,52 | 1062,08 | 0,2186 |

AIC = Akaike Information Criterion; BIC = Bayesian Information Criterion.
