# Supplementary material for: Spatial distribution of the time between diagnosis and treatment of breast cancer among women in Brazil
Source: PLoS One. 2026 Oct 28;21(10):e0361469. doi: 10.1371/journal.pone.0361469 (PMC13716208; doi:10.1371/journal.pone.0361469)
Supplement: Supplementary file 2 [file pone.0361469.s002.docx]

| **Year** | **Total breast cancer cases** | **Treatment ≤ 60 days (n)** | **Percentual treatment ≤ 60 days** |
| --- | --- | --- | --- |
| 2013 | 24,795 | 12,950 | 52.2% |
| 2014 | 26,771 | 13,664 | 51.0% |
| 2015 | 26,782 | 13,598 | 50.8% |
| 2016 | 28,398 | 14,331 | 50.5% |
| 2017 | 28,818 | 14,025 | 48.7% |
| 2018 | 28,450 | 13,242 | 46.5% |
| 2019 | 28,378 | 12,295 | 43.3% |

S2 Table. Distribution of included breast cancer cases and treatment initiation within 60 days by year, Brazil, 2013-2019.
