# Supplementary material for: Spatial distribution of the time between diagnosis and treatment of breast cancer among women in Brazil
Source: PLoS One. 2026 Oct 28;21(10):e0361469. doi: 10.1371/journal.pone.0361469 (PMC13716208; doi:10.1371/journal.pone.0361469)
Supplement: Supplementary file 3 [file pone.0361469.s003.docx]

S3 Table. Distribution of included breast cancer cases and treatment initiation within 60 days by Intermediate Region of Urban Articulation. Brazil. 2013-2019.

| **IRUA**  **Code** | **IRUA Name** | **Total cases** | **Treatment ≤60 days (n)** | **Treatment ≤60 days (%)** |
| --- | --- | --- | --- | --- |
| 1101 | Porto Velho | 603 | 225 | 37.3 |
| 1102 | Ji-Paraná | 610 | 248 | 40.7 |
| 1201 | Rio Branco | 267 | 100 | 37.5 |
| 1202 | Cruzeiro do Sul | 44 | 13 | 29.5 |
| 1301 | Manaus | 1,674 | 608 | 36.3 |
| 1302 | Tefé | 54 | 20 | 37.0 |
| 1303 | Lábrea | 42 | 16 | 38.1 |
| 1304 | Parintins | 93 | 31 | 33.3 |
| 1401 | Boa Vista | 123 | 44 | 35.8 |
| 1402 | Rorainópolis - Caracaraí | 14 | 6 | 42.9 |
| 1501 | Belém | 1,826 | 604 | 33.1 |
| 1502 | Castanhal | 472 | 170 | 36.0 |
| 1503 | Marabá | 199 | 75 | 37.7 |
| 1504 | Redenção | 105 | 33 | 31.4 |
| 1505 | Santarém | 412 | 162 | 39.3 |
| 1506 | Altamira | 68 | 13 | 19.1 |
| 1507 | Breves | 111 | 33 | 29.7 |
| 1601 | Macapá | 232 | 120 | 51.7 |
| 1602 | Oiapoque - Porto Grande | 14 | 6 | 42.9 |
| 1701 | Palmas | 375 | 232 | 61.9 |
| 1702 | Araguaína | 429 | 307 | 71.6 |
| 1703 | Gurupi | 127 | 71 | 55.9 |
| 2101 | São Luís | 2,485 | 1,585 | 63.8 |
| 2102 | Santa Inês - Bacabal | 382 | 254 | 66.5 |
| 2103 | Caxias | 206 | 65 | 31.6 |
| 2104 | Presidente Dutra | 121 | 72 | 59.5 |
| 2105 | Imperatriz | 473 | 271 | 57.3 |
| 2201 | Teresina | 1,192 | 582 | 48.8 |
| 2202 | Parnaíba | 211 | 90 | 42.7 |
| 2203 | Picos | 266 | 136 | 51.1 |
| 2204 | São Raimundo Nonato | 56 | 25 | 44.6 |
| 2205 | Corrente - Bom Jesus | 73 | 40 | 54.8 |
| 2206 | Floriano | 129 | 63 | 48.8 |
| 2301 | Fortaleza | 4,742 | 2,835 | 59.8 |
| 2302 | Quixadá | 598 | 300 | 50.2 |
| 2303 | Iguatu | 221 | 112 | 50.7 |
| 2304 | Juazeiro do Norte | 1,181 | 452 | 38.3 |
| 2305 | Crateús | 235 | 111 | 47.2 |
| 2306 | Sobral | 821 | 464 | 56.5 |
| 2401 | Natal | 2,524 | 1,964 | 77.8 |
| 2402 | Caicó | 363 | 272 | 74.9 |
| 2403 | Mossoró | 1,212 | 920 | 75.9 |
| 2501 | João Pessoa | 2,593 | 1,062 | 41.0 |
| 2502 | Campina Grande | 1,552 | 507 | 32.7 |
| 2503 | Patos | 683 | 272 | 39.8 |
| 2504 | Sousa - Cajazeiras | 328 | 152 | 46.3 |
| 2601 | Recife | 6,538 | 4,333 | 66.3 |
| 2602 | Caruaru | 1,529 | 857 | 56.0 |
| 2603 | Serra Talhada | 316 | 193 | 61.1 |
| 2604 | Petrolina | 672 | 346 | 51.5 |
| 2701 | Maceió | 1,971 | 1,082 | 54.9 |
| 2702 | Arapiraca | 973 | 539 | 55.4 |
| 2801 | Aracaju | 1,451 | 769 | 53.0 |
| 2802 | Itabaiana | 533 | 271 | 50.8 |
| 2901 | Salvador | 4,261 | 2,227 | 52.3 |
| 2902 | Santo Antônio de Jesus | 681 | 332 | 48.8 |
| 2903 | Ilhéus – Itabuna | 1,005 | 430 | 42.8 |
| 2904 | Vitória da Conquista | 784 | 289 | 36.9 |
| 2905 | Guanambi | 224 | 107 | 47.8 |
| 2906 | Barreiras | 208 | 82 | 39.4 |
| 2907 | Irecê | 330 | 128 | 38.8 |
| 2908 | Juazeiro | 602 | 309 | 51.3 |
| 2909 | Paulo Afonso | 432 | 188 | 43.5 |
| 2910 | Feira de Santana | 1,824 | 1,092 | 59.9 |
| 3101 | Belo Horizonte | 7,828 | 4,195 | 53.6 |
| 3102 | Montes Claros | 1,257 | 777 | 61.8 |
| 3103 | Teófilo Otoni | 810 | 295 | 36.4 |
| 3104 | Governador Valadares | 661 | 367 | 55.5 |
| 3105 | Ipatinga | 1,393 | 572 | 41.1 |
| 3106 | Juíz de Fora | 3,023 | 2,191 | 72.5 |
| 3107 | Barbacena | 1,017 | 621 | 61.1 |
| 3108 | Varginha | 2,225 | 1,659 | 74.6 |
| 3109 | Pouso Alegre | 1,612 | 805 | 49.9 |
| 3110 | Uberaba | 972 | 495 | 50.9 |
| 3111 | Uberlândia | 1,100 | 468 | 42.5 |
| 3112 | Patos de Minas | 498 | 156 | 31.3 |
| 3113 | Divinópolis | 1,717 | 640 | 37.3 |
| 3201 | Vitória | 2,596 | 1,912 | 73.7 |
| 3202 | São Mateus | 481 | 331 | 68.8 |
| 3203 | Colatina | 493 | 299 | 60.6 |
| 3204 | Cachoeiro do Itapemirim | 965 | 742 | 76.9 |
| 3301 | Rio de Janeiro | 12,080 | 2,005 | 16.6 |
| 3302 | Volta Redonda - Barra Mansa | 159 | 40 | 25.2 |
| 3303 | Petrópolis | 1,204 | 269 | 22.3 |
| 3304 | Campos dos Goytacazes | 1,401 | 643 | 45.9 |
| 3305 | Macaé - Rio das Ostras - Cabo Frio | 685 | 173 | 25.3 |
| 3501 | São Paulo | 26,099 | 10,353 | 39.7 |
| 3502 | Sorocaba | 2,556 | 862 | 33.7 |
| 3503 | Bauru | 2,344 | 1,343 | 57.3 |
| 3504 | Marília | 1,764 | 959 | 54.4 |
| 3505 | Presidente Prudente | 1,003 | 420 | 41.9 |
| 3506 | Araçatuba | 1,321 | 404 | 30.6 |
| 3507 | São José do Rio Preto | 2,683 | 963 | 35.9 |
| 3508 | Ribeirão Preto | 3,463 | 1,570 | 45.3 |
| 3509 | Araraquara | 1,205 | 673 | 55.9 |
| 3510 | Campinas | 6,937 | 3,007 | 43.3 |
| 3511 | São José dos Campos | 1,632 | 799 | 49.0 |
| 4101 | Curitiba | 4,088 | 2,751 | 67.3 |
| 4102 | Guarapuava | 482 | 344 | 71.4 |
| 4103 | Cascavel | 2,488 | 2,116 | 85.0 |
| 4104 | Maringá | 1,845 | 1,413 | 76.6 |
| 4105 | Londrina | 2,459 | 1,344 | 54.7 |
| 4106 | Ponta Grossa | 645 | 333 | 51.6 |
| 4201 | Florianópolis | 1,334 | 501 | 37.6 |
| 4202 | Criciúma | 1,340 | 730 | 54.5 |
| 4203 | Lages | 451 | 208 | 46.1 |
| 4204 | Chapecó | 1,412 | 480 | 34.0 |
| 4205 | Caçador | 149 | 77 | 51.7 |
| 4206 | Joinville | 1,680 | 837 | 49.8 |
| 4207 | Blumenau | 1,241 | 389 | 31.3 |
| 4301 | Porto Alegre | 4,774 | 2,913 | 61.0 |
| 4302 | Pelotas | 2,022 | 1,100 | 54.4 |
| 4303 | Santa Maria | 1,288 | 622 | 48.3 |
| 4304 | Uruguaiana | 669 | 421 | 62.9 |
| 4305 | Ijuí | 1,354 | 595 | 43.9 |
| 4306 | Passo Fundo | 2,638 | 1,580 | 59.9 |
| 4307 | Caxias do Sul | 1,189 | 809 | 68.0 |
| 4308 | Santa Cruz do Sul - Lajeado | 1,331 | 856 | 64.3 |
| 5001 | Campo Grande | 1,304 | 615 | 47.2 |
| 5002 | Dourados | 496 | 227 | 45.8 |
| 5003 | Corumbá | 236 | 119 | 50.4 |
| 5101 | Cuiabá | 969 | 433 | 44.7 |
| 5102 | Cáceres | 102 | 28 | 27.5 |
| 5103 | Sinop | 351 | 156 | 44.4 |
| 5104 | Barra do Garças | 107 | 32 | 29.9 |
| 5105 | Rondonópolis | 214 | 82 | 38.3 |
| 5201 | Goiânia | 508 | 275 | 54.1 |
| 5202 | Itumbiara | 211 | 55 | 26.1 |
| 5203 | Rio Verde | 375 | 68 | 18.1 |
| 5204 | São Luís de Montes Belos - Iporá | 42 | 18 | 42.9 |
| 5205 | Porangatu - Uruaçu | 145 | 56 | 38.6 |
| 5206 | Luziânia - Águas Lindas de Goiás | 389 | 200 | 51.4 |
| 5301 | Distrito Federal | 2,002 | 995 | 49.7 |

IRUA: Intermediate Region of Urban Articulation.
