# Supplementary material for: Spatial distribution of the time between diagnosis and treatment of breast cancer among women in Brazil
Source: PLoS One. 2026 Oct 28;21(10):e0361469. doi: 10.1371/journal.pone.0361469 (PMC13716208; doi:10.1371/journal.pone.0361469)
Supplement: Supplementary file 4 [file pone.0361469.s004.docx]

S4 Table. Distribution of included breast cancer cases by year and Intermediate Region of Urban Articulation, Brazil, 2013-2019.

| **IRUA**  **Code** | **IRUA Name** | **2013** | **2014** | **2015** | **2016** | **2017** | **2018** | **2019** | **Total** |
| --- | --- | --- | --- | --- | --- | --- | --- | --- | --- |
| 1101 | Porto Velho | 48 | 30 | 42 | 121 | 86 | 129 | 147 | 603 |
| 1102 | Ji-Paraná | 39 | 34 | 44 | 210 | 60 | 94 | 129 | 610 |
| 1201 | Rio Branco | 27 | 28 | 26 | 30 | 48 | 58 | 50 | 267 |
| 1202 | Cruzeiro do Sul | 4 | 6 | 5 | 9 | 4 | 9 | 7 | 44 |
| 1301 | Manaus | 198 | 192 | 232 | 258 | 225 | 290 | 279 | 1,674 |
| 1302 | Tefé | 2 | 11 | 6 | 8 | 5 | 12 | 10 | 54 |
| 1303 | Lábrea | 1 | 2 | 6 | 6 | 1 | 13 | 13 | 42 |
| 1304 | Parintins | 8 | 13 | 15 | 8 | 8 | 26 | 15 | 93 |
| 1401 | Boa Vista | 17 | 11 | 9 | 23 | 30 | 27 | 6 | 123 |
| 1402 | Rorainópolis - Caracaraí | 2 | 0 | 2 | 1 | 5 | 4 | 0 | 14 |
| 1501 | Belém | 196 | 233 | 206 | 270 | 275 | 327 | 319 | 1,826 |
| 1502 | Castanhal | 52 | 61 | 57 | 60 | 80 | 76 | 86 | 472 |
| 1503 | Marabá | 27 | 31 | 24 | 27 | 40 | 26 | 24 | 199 |
| 1504 | Redenção | 2 | 9 | 18 | 11 | 15 | 30 | 20 | 105 |
| 1505 | Santarém | 58 | 49 | 51 | 70 | 37 | 56 | 91 | 412 |
| 1506 | Altamira | 6 | 10 | 7 | 9 | 15 | 14 | 7 | 68 |
| 1507 | Breves | 10 | 15 | 10 | 26 | 17 | 14 | 19 | 111 |
| 1601 | Macapá | 16 | 29 | 36 | 48 | 39 | 14 | 50 | 232 |
| 1602 | Oiapoque - Porto Grande | 1 | 1 | 5 | 2 | 1 | 1 | 3 | 14 |
| 1701 | Palmas | 34 | 48 | 49 | 60 | 47 | 66 | 71 | 375 |
| 1702 | Araguaína | 36 | 52 | 63 | 52 | 67 | 79 | 80 | 429 |
| 1703 | Gurupi | 14 | 20 | 18 | 12 | 26 | 20 | 17 | 127 |
| 2101 | São Luís | 324 | 298 | 374 | 382 | 366 | 376 | 365 | 2,485 |
| 2102 | Santa Inês - Bacabal | 41 | 41 | 55 | 53 | 60 | 74 | 58 | 382 |
| 2103 | Caxias | 38 | 17 | 27 | 48 | 29 | 26 | 21 | 206 |
| 2104 | Presidente Dutra | 10 | 15 | 16 | 18 | 22 | 23 | 17 | 121 |
| 2105 | Imperatriz | 54 | 41 | 40 | 54 | 96 | 88 | 100 | 473 |
| 2201 | Teresina | 150 | 142 | 171 | 167 | 163 | 164 | 235 | 1,192 |
| 2202 | Parnaíba | 37 | 29 | 38 | 31 | 26 | 21 | 29 | 211 |
| 2203 | Picos | 51 | 16 | 40 | 48 | 23 | 39 | 49 | 266 |
| 2204 | São Raimundo Nonato | 4 | 6 | 8 | 8 | 8 | 10 | 12 | 56 |
| 2205 | Corrente - Bom Jesus | 7 | 6 | 10 | 12 | 10 | 15 | 13 | 73 |
| 2206 | Floriano | 17 | 9 | 14 | 22 | 9 | 29 | 29 | 129 |
| 2301 | Fortaleza | 660 | 714 | 671 | 706 | 658 | 663 | 670 | 4,742 |
| 2302 | Quixadá | 56 | 69 | 67 | 81 | 88 | 124 | 113 | 598 |
| 2303 | Iguatu | 20 | 29 | 24 | 44 | 26 | 35 | 43 | 221 |
| 2304 | Juazeiro do Norte | 182 | 155 | 132 | 174 | 156 | 192 | 190 | 1,181 |
| 2305 | Crateús | 19 | 20 | 28 | 47 | 44 | 33 | 44 | 235 |
| 2306 | Sobral | 117 | 108 | 87 | 116 | 112 | 99 | 182 | 821 |
| 2401 | Natal | 431 | 477 | 466 | 411 | 411 | 313 | 15 | 2,524 |
| 2402 | Caicó | 71 | 55 | 75 | 62 | 53 | 46 | 1 | 363 |
| 2403 | Mossoró | 167 | 206 | 191 | 204 | 176 | 162 | 106 | 1,212 |
| 2501 | João Pessoa | 296 | 377 | 361 | 372 | 382 | 431 | 374 | 2,593 |
| 2502 | Campina Grande | 172 | 166 | 197 | 229 | 237 | 254 | 297 | 1,552 |
| 2503 | Patos | 94 | 93 | 105 | 116 | 120 | 104 | 51 | 683 |
| 2504 | Sousa - Cajazeiras | 41 | 43 | 43 | 53 | 55 | 53 | 40 | 328 |
| 2601 | Recife | 881 | 907 | 858 | 957 | 1,023 | 1,023 | 889 | 6,538 |
| 2602 | Caruaru | 229 | 267 | 276 | 270 | 186 | 169 | 132 | 1,529 |
| 2603 | Serra Talhada | 47 | 59 | 47 | 52 | 40 | 44 | 27 | 316 |
| 2604 | Petrolina | 101 | 110 | 109 | 108 | 84 | 118 | 42 | 672 |
| 2701 | Maceió | 273 | 286 | 213 | 273 | 325 | 288 | 313 | 1,971 |
| 2702 | Arapiraca | 119 | 130 | 110 | 151 | 170 | 127 | 166 | 973 |
| 2801 | Aracaju | 186 | 141 | 90 | 205 | 235 | 262 | 332 | 1,451 |
| 2802 | Itabaiana | 63 | 46 | 46 | 74 | 72 | 98 | 134 | 533 |
| 2901 | Salvador | 561 | 626 | 555 | 655 | 672 | 728 | 464 | 4,261 |
| 2902 | Santo Antônio de Jesus | 104 | 107 | 117 | 88 | 94 | 99 | 72 | 681 |
| 2903 | Ilhéus – Itabuna | 142 | 131 | 117 | 137 | 222 | 134 | 122 | 1,005 |
| 2904 | Vitória da Conquista | 117 | 162 | 141 | 142 | 137 | 46 | 39 | 784 |
| 2905 | Guanambi | 30 | 36 | 39 | 36 | 38 | 32 | 13 | 224 |
| 2906 | Barreiras | 14 | 32 | 35 | 31 | 34 | 37 | 25 | 208 |
| 2907 | Irecê | 42 | 40 | 51 | 52 | 62 | 49 | 34 | 330 |
| 2908 | Juazeiro | 92 | 84 | 82 | 111 | 90 | 91 | 52 | 602 |
| 2909 | Paulo Afonso | 57 | 69 | 54 | 61 | 84 | 74 | 33 | 432 |
| 2910 | Feira de Santana | 256 | 283 | 271 | 287 | 235 | 271 | 221 | 1,824 |
| 3101 | Belo Horizonte | 791 | 937 | 973 | 1,194 | 1,260 | 1,258 | 1,415 | 7,828 |
| 3102 | Montes Claros | 159 | 245 | 160 | 183 | 121 | 173 | 216 | 1,257 |
| 3103 | Teófilo Otoni | 94 | 103 | 89 | 92 | 126 | 135 | 171 | 810 |
| 3104 | Governador Valadares | 100 | 134 | 87 | 93 | 79 | 87 | 81 | 661 |
| 3105 | Ipatinga | 207 | 181 | 198 | 213 | 170 | 190 | 234 | 1,393 |
| 3106 | Juíz de Fora | 483 | 438 | 419 | 406 | 417 | 379 | 481 | 3,023 |
| 3107 | Barbacena | 111 | 133 | 154 | 136 | 162 | 162 | 159 | 1,017 |
| 3108 | Varginha | 278 | 269 | 306 | 294 | 315 | 363 | 400 | 2,225 |
| 3109 | Pouso Alegre | 219 | 255 | 194 | 212 | 229 | 247 | 256 | 1,612 |
| 3110 | Uberaba | 46 | 172 | 133 | 142 | 128 | 171 | 180 | 972 |
| 3111 | Uberlândia | 138 | 152 | 121 | 143 | 184 | 182 | 180 | 1,100 |
| 3112 | Patos de Minas | 36 | 57 | 52 | 86 | 75 | 94 | 98 | 498 |
| 3113 | Divinópolis | 203 | 270 | 218 | 243 | 261 | 249 | 273 | 1,717 |
| 3201 | Vitória | 294 | 337 | 357 | 362 | 403 | 404 | 439 | 2,596 |
| 3202 | São Mateus | 49 | 53 | 62 | 81 | 66 | 62 | 108 | 481 |
| 3203 | Colatina | 30 | 71 | 85 | 87 | 67 | 70 | 83 | 493 |
| 3204 | Cachoeiro do Itapemirim | 106 | 144 | 128 | 154 | 163 | 127 | 143 | 965 |
| 3301 | Rio de Janeiro | 1,497 | 1,500 | 1,525 | 1,685 | 2,058 | 1,937 | 1,878 | 12,080 |
| 3302 | Volta Redonda - Barra Mansa | 16 | 65 | 40 | 12 | 9 | 11 | 6 | 159 |
| 3303 | Petrópolis | 149 | 185 | 168 | 175 | 171 | 153 | 203 | 1,204 |
| 3304 | Campos dos Goytacazes | 158 | 200 | 166 | 181 | 221 | 247 | 228 | 1,401 |
| 3305 | Macaé - Rio das Ostras - Cabo Frio | 96 | 102 | 146 | 101 | 157 | 39 | 44 | 685 |
| 3501 | São Paulo | 3,884 | 4,123 | 3,897 | 3,715 | 3,387 | 3,511 | 3,582 | 26,099 |
| 3502 | Sorocaba | 338 | 382 | 395 | 348 | 353 | 348 | 392 | 2,556 |
| 3503 | Bauru | 321 | 335 | 331 | 360 | 365 | 342 | 290 | 2,344 |
| 3504 | Marília | 252 | 261 | 229 | 269 | 270 | 279 | 204 | 1,764 |
| 3505 | Presidente Prudente | 98 | 121 | 150 | 149 | 141 | 144 | 200 | 1,003 |
| 3506 | Araçatuba | 192 | 189 | 178 | 195 | 185 | 198 | 184 | 1,321 |
| 3507 | São José do Rio Preto | 312 | 368 | 378 | 376 | 420 | 399 | 430 | 2,683 |
| 3508 | Ribeirão Preto | 485 | 449 | 451 | 474 | 556 | 474 | 574 | 3,463 |
| 3509 | Araraquara | 207 | 219 | 210 | 193 | 126 | 127 | 123 | 1,205 |
| 3510 | Campinas | 986 | 1,019 | 1,065 | 1,203 | 1,066 | 748 | 850 | 6,937 |
| 3511 | São José dos Campos | 258 | 296 | 264 | 162 | 207 | 260 | 185 | 1,632 |
| 4101 | Curitiba | 544 | 512 | 471 | 529 | 628 | 730 | 674 | 4,088 |
| 4102 | Guarapuava | 67 | 62 | 63 | 70 | 77 | 80 | 63 | 482 |
| 4103 | Cascavel | 381 | 304 | 327 | 364 | 340 | 392 | 380 | 2,488 |
| 4104 | Maringá | 245 | 287 | 300 | 303 | 304 | 202 | 204 | 1,845 |
| 4105 | Londrina | 297 | 305 | 336 | 355 | 376 | 362 | 428 | 2,459 |
| 4106 | Ponta Grossa | 97 | 67 | 47 | 108 | 131 | 86 | 109 | 645 |
| 4201 | Florianópolis | 202 | 171 | 167 | 193 | 189 | 188 | 224 | 1,334 |
| 4202 | Criciúma | 111 | 119 | 173 | 200 | 211 | 294 | 232 | 1,340 |
| 4203 | Lages | 28 | 74 | 74 | 66 | 76 | 76 | 57 | 451 |
| 4204 | Chapecó | 184 | 197 | 227 | 220 | 197 | 226 | 161 | 1,412 |
| 4205 | Caçador | 12 | 20 | 45 | 31 | 36 | 2 | 3 | 149 |
| 4206 | Joinville | 199 | 204 | 245 | 238 | 263 | 250 | 281 | 1,680 |
| 4207 | Blumenau | 197 | 212 | 245 | 131 | 83 | 135 | 238 | 1,241 |
| 4301 | Porto Alegre | 477 | 544 | 809 | 690 | 697 | 711 | 846 | 4,774 |
| 4302 | Pelotas | 229 | 357 | 300 | 278 | 308 | 296 | 254 | 2,022 |
| 4303 | Santa Maria | 160 | 131 | 166 | 212 | 211 | 208 | 200 | 1,288 |
| 4304 | Uruguaiana | 113 | 152 | 138 | 88 | 112 | 34 | 32 | 669 |
| 4305 | Ijuí | 152 | 233 | 178 | 215 | 174 | 194 | 208 | 1,354 |
| 4306 | Passo Fundo | 276 | 396 | 388 | 395 | 386 | 401 | 396 | 2,638 |
| 4307 | Caxias do Sul | 154 | 178 | 239 | 183 | 188 | 58 | 189 | 1,189 |
| 4308 | Santa Cruz do Sul - Lajeado | 140 | 167 | 145 | 192 | 235 | 222 | 230 | 1,331 |
| 5001 | Campo Grande | 155 | 181 | 195 | 187 | 235 | 200 | 151 | 1,304 |
| 5002 | Dourados | 37 | 67 | 42 | 39 | 92 | 99 | 120 | 496 |
| 5003 | Corumbá | 20 | 21 | 26 | 33 | 37 | 57 | 42 | 236 |
| 5101 | Cuiabá | 203 | 163 | 127 | 140 | 119 | 138 | 79 | 969 |
| 5102 | Cáceres | 25 | 10 | 23 | 9 | 11 | 14 | 10 | 102 |
| 5103 | Sinop | 48 | 45 | 46 | 44 | 66 | 61 | 41 | 351 |
| 5104 | Barra do Garças | 14 | 10 | 14 | 21 | 18 | 17 | 13 | 107 |
| 5105 | Rondonópolis | 38 | 30 | 48 | 20 | 40 | 23 | 15 | 214 |
| 5201 | Goiânia | 67 | 43 | 37 | 122 | 128 | 68 | 43 | 508 |
| 5202 | Itumbiara | 22 | 27 | 29 | 27 | 37 | 40 | 29 | 211 |
| 5203 | Rio Verde | 54 | 49 | 39 | 57 | 67 | 54 | 55 | 375 |
| 5204 | São Luís de Montes Belos - Iporá | 3 | 0 | 2 | 9 | 16 | 3 | 9 | 42 |
| 5205 | Porangatu - Uruaçu | 23 | 10 | 22 | 33 | 37 | 20 | 0 | 145 |
| 5206 | Luziânia - Águas Lindas de Goiás | 17 | 30 | 68 | 70 | 77 | 69 | 58 | 389 |
| 5301 | Distrito Federal | 39 | 94 | 269 | 338 | 434 | 421 | 407 | 2,002 |

IRUA: Intermediate Region of Urban Articulation.
